# Pediatric Fungal Septic Arthritis Following Intra-articular Administration of the Two-Month Vaccination: A Case Report

**DOI:** 10.7759/cureus.73777

**Published:** 2024-11-15

**Authors:** Amin G Gronfula, Thamer H Alsharif, Raef F Alamri, Abdulellah L Almoutairi, Ahmed Khawjah, Ayman A Alzahrani, Zeyad M Bukhari, Fahad Abduljabbar

**Affiliations:** 1 Orthopedic Surgery, Al-Noor Specialist Hospital, Makkah, SAU; 2 Medicine, Royal College of Surgeons, Dublin, IRL; 3 Orthopedics and Traumatology, Al-Noor Specialist Hospital, Makkah, SAU; 4 General Surgery, Galway University Hospital, Galway, IRL; 5 Orthopedics, Al-Noor Specialist Hospital, Makkah, SAU; 6 Orthopedic Surgery, King Abdulaziz University, Jeddah, SAU

**Keywords:** hip, neonate, orthopedic, septic, surgery

## Abstract

The majority of pediatric fungal septic arthritis patients are infants. Risk factors include prematurity and neonatal septicemia with prolonged hospitalization. Here, we present a case of a two-month-old male infant, preterm at 28 weeks and NICU graduate. He was transferred to King Abdulaziz University Hospital (KAUH) in Jeddah, Saudi Arabia due to left hip septic Candida arthritis that was unresponsive to antifungal treatment in an outside hospital. Initially, he was administered the two-month scheduled vaccines inadvertently in the intra-articular space of the left hip. Three days later, he developed candidemia and symptoms of septic arthritis in the left hip. Joint aspiration grew Candida and he was then commenced on anti-fungal treatment. In our hospital, he was vitally stable and febrile. Examination showed erythema, warmth, and severe tenderness, with pain and reduced range of motion of the left hip. Inflammatory markers were increased. X-ray of the left hip was unremarkable. MRI with contrast showed mild left hip effusion associated with synovial enhancement and soft tissue edema and enhancement. Incision and drainage was done after which he received vancomycin and meropenem for four weeks along with fluconazole for eight weeks. A back slab was applied for four weeks. The patient achieved successful recovery upon completion of the treatment and incision and drainage. To our knowledge, this is the first reported case of fungal septic arthritis as a consequence of intra-articular vaccination administration. This case highlights the importance of considering fungi as an etiology of pediatric septic arthritis, particularly in patients with the aforementioned risk factors.

## Introduction

Infants account for around 85% of pediatric fungal arthritis cases, a rare but important etiology that should be recognized and managed early [1]. The most common causative organisms of fungal septic arthritis are Candida and Aspergillus [2]. Candida arthritis tends to present late with milder symptoms compared to other etiologies of septic arthritis, usually manifesting with joint or extremity swelling and reduced range of motion [3]. However, in approximately 70-80% of patients with fungal arthritis, it can result in osteomyelitis [4]. Candida mostly spreads to joints via the hematogenous route in the case of candidemia, but can also occur following exogenous inoculation [5].

In this paper, we report a case of a two-month-old infant who acquired fungal septic arthritis of the hip joint following inadvertent intra-articular administration of the two-month scheduled vaccinations in Saudi Arabia. There are a few case reports of pediatric septic arthritis following vaccination; however, to the best of our knowledge, this is the first case of Candida septic arthritis in an infant following vaccination [6-9].

## Case presentation

A two-month-old male infant, preterm at 28 weeks and NICU graduate, was transferred to our emergency department due to non-responsive left hip fungal septic arthritis. The patient's main complaint was the limited range of motion in the left hip after receiving his two-month vaccination. On examination, extreme pain on movement and palpation, no range of motion on active and passive movement of the left hip, extreme tenderness and hotness, and redness all over the left hip are noticed. The patient was vitally stable with a temperature of 39 degrees. The pain did not respond to the NSAIDS. The patient is a NICU graduate, preterm at 28 weeks, who was hospitalized for 30 days due to respiratory distress syndrome (RDS) and received two doses of surfactant. The patient was intubated and received respiratory support due to bronchopulmonary dysplasia. The musculoskeletal symptoms started three days after receiving his two-month vaccination. The injection was given incidentally to the patient intra-articularly instead of intra-muscularly, which led to septic arthritis.

**Figure 1:**
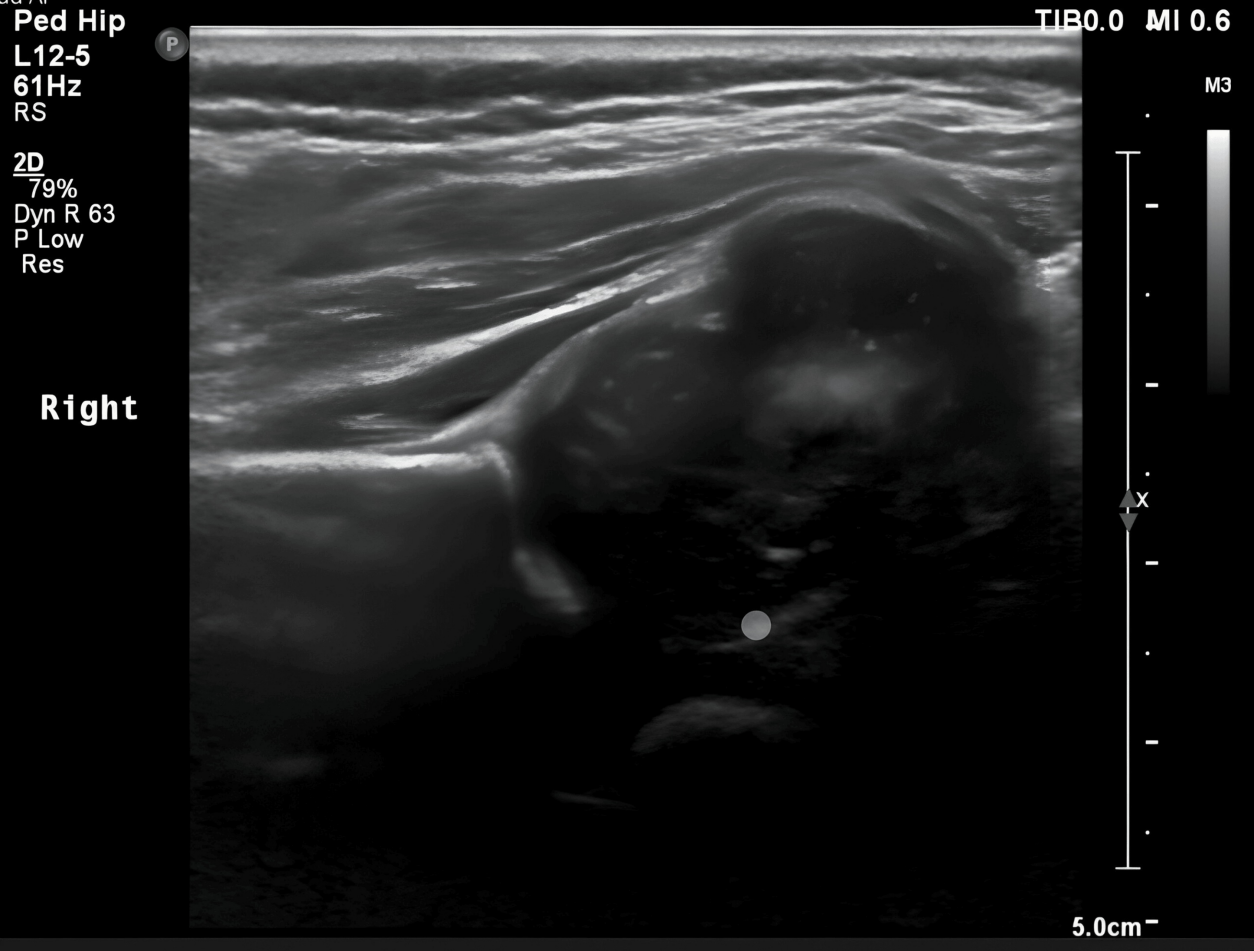
Ultrasound that identifies the collection

Inflammatory markers were increased (ESR: 36, CRP: 20). Blood, CSF, and urine cultures were negative. Joint aspiration with incision and drainage was done and showed Candida in the previous hospital. Meropenem, vancomycin, and fluconazole were commenced, and further imaging was requested. Ultrasound image of the hip showed significant purulent fluid collection in the joint space before aspiration (Figure 1).

Meropenem and vancomycin were given intravenously for four weeks and fluconazole for eight weeks. The back slab was placed on the patient for a total of four weeks, changing the back slab every three days with a day off in between, to allow positioning of the hip in full extension. A complete improvement in the range of motion was noticed after the removal of the back slab and the completion of antibiotics. The patient was discharged on fluconazole for four weeks. Physiotherapy was commenced, and an OPD appointment was given after one month.

## Discussion

Septic arthritis is a serious emergency among the pediatric population that could lead to life-threatening events, articular cartilage destruction, early onset arthritis, and joint deformity. The joint space is the mainstay for the microorganism through direct inoculation or homogenous spread, leading to the spread of the infection. There are various risk factors for septic arthritis, including intra-articular injections, immunodeficiency, joint surgery, rheumatoid arthritis, and joint aspiration [10-12]. The hip is thought to be one of the most sensitive sites that could be permanently affected by septic arthritis due to its anatomical position. The hips are one of the most common sites to be involved in the pediatric population, with a prevalence of 32-40% [13-15]. Age is a significant risk factor in this population, as most cases of septic arthritis (33%-50%) arise in children under two years of age, and the type of most common causative organisms changes among different ages [16-21].

The diagnosis of septic arthritis in the pediatric population is complex but has significant importance due to its variation in presentation. Fever, inability to bear weight, and neonates usually present with hip flexion and abduction in internal rotation and fixation of hip joint position [22]. The diagnostic algorithm usually starts after a clinical examination and history. After suspecting septic arthritis, the clinician has to look at the erythrocyte sedimentation rate, c-reactive protein, and white blood cells. If the CRP is >20 mg/L and the ESR is higher than 20 mm/h, then joint aspiration and empiric antibiotic treatments should be commenced. If they are lower than the reported values, then the patient should be monitored and a repeat examination should be done. The sample acquired by joint aspiration should be sent with blood cultures to bacteriology [23]. Cultures are not a strong indicator as they can give false negatives (30-70% negative cultures in all cases) [24,25]. Joint effusion, or chronic osteomyelitis, can be detected by X-ray but, MRI is superior to X-ray when detecting osteomyelitis, abscesses, or effusion [26].

Treatment of septic arthritis in the pediatric population differs due to its variety and severity. The mainstay of treatment that should be initiated once a diagnosis is made is irrigation and debridement followed by empirical antibiotics. Dexamethasone administration is thought to aid in the reduction of hospital stays due to its ability to reduce inflammation; pain relief medication should also be initiated, and joint stabilization should be considered [27]. CRP monitoring is beneficial to measure improvement. Antibiotics can be safely stopped once CRP reaches a level below 20 mg/L, which indicates recovery [23,28]. Repeat debridement might be necessary in case of persistent septic arthritis despite appropriate anti-fungal treatment [29]. Early detection and treatment of septic arthritis can lead to a better prognosis and the avoidance of life-long outcomes. The following table provides a comparison between fungal septic arthritis with bacterial septic arthritis (Table 1).

**Table 1:** Comparison between fungal septic arthritis and bacterial septic arthritis Credits: Amin G. Gronfula

Characteristic	Bacterial septic arthritis	Fungal septic arthritis
Prevalence [29-30]	Most common	Rare
Most common pathogen [29-30]	Staphylococcus aureus	Candida albicans
Onset of symptoms [31]	Earlier onset of symptoms	More insidious
Risk factors [11-13,30]	Intra-articular injections, immunodeficiency, joint surgery, rheumatoid arthritis, and joint aspiration	Pre-term births, low birth weights, NICU patient, in addition to the aforementioned risk factors in bacterial septic arthritis
Diagnosis [29-30]	CRP >20 mg/L; ESR >20 mm/h; synovial fluid with WBC of >50000/μL; positive synovial fluid culture (may be negative in 30-70% of cultures); X-ray/MRI	Similar to bacterial septic arthritis. fungal septic arthritis should be suspected in patients with negative bacterial culture results with a high clinical suspicion for septic arthritis
Duration of culture growth [31]	Days	Can take up to four weeks
Clinical features [22,29]	Erythema, warmth, tenderness, pain, and reduced range of motion. Swelling neonates usually present with hip flexion and abduction in internal rotation and fixation of hip joint position	Erythema, warmth, tenderness, pain, and reduced range of motion. Swelling neonates usually present with hip flexion and abduction in internal rotation and fixation of hip joint position
Treatment [29-31]	Irrigation and debridement, followed by empiric antibiotics. Antibiotics of choice are first-generation cephalosporins and clindamycin. In patients with MRSA, vancomycin is preferred	Irrigation and debridement, followed by empiric antibiotics. Antifungal drug of choice is amphotericin B or fluconazole

## Conclusions

Fungal septic arthritis in the pediatric population is a rare yet serious emergency that requires early detection and intervention to avoid permanent joint damage and life-threatening complications. Fungal septic arthritis should be suspected in patients with high clinical suspicion for septic arthritis despite negative synovial fluid culture results. Treatment involves irrigation and debridement, followed by empiric anti-fungal treatment. Monitoring is then done with CRP to detect recovery. A repeat debridement might be needed in persistent infection.
